# CT-based Radiomics of Intratumoral and Peritumoral Regions to Predict the Recurrence Risk in Patients with Non-muscle-invasive Bladder Cancer within Two Years after TURBT

**DOI:** 10.2174/0115734056350444250418075406

**Published:** 2025-05-26

**Authors:** Ting Cao, Na Li, Chuanchao Guo, Hepeng Zhang, Lihua Chen, Ke Wu, Lisha Liang, Ximing Wang, Wen Shen

**Affiliations:** 1 Department of Radiology, First Central Clinical College, Tianjin Medical University, Tianjin, China; 2 Department of Radiology, The People’s Hospital of Zhangqiu Area, Jinan, China; 3 Department of Radiology, The Affiliated Taian City Central Hospital of Qingdao University, Taian, China; 4 Department of Urology Surgery, The Affiliated Taian City Central Hospital of Qingdao University, Taian, China; 5 Department of Radiology, Tianjin First Central Hospital, School of Medicine, Nankai University, Tianjin, China; 6 Department of Radiology, Shandong Provincial Hospital Affiliated to Shandong First Medical University, Jinan, China

**Keywords:** CT, Radiomics, Machine learning, Recurrence, Non-muscle-invasive bladder cancer, Transurethral resection of bladder tumor, Bladder cancer, TME

## Abstract

**Background:**

Predicting the recurrence risk of NMIBC after TURBT is crucial for individualized clinical treatment.

**Objective:**

The objective of this study is to evaluate the ability of radiomic feature analysis of intratumoral and peritumoral regions based on computed tomography (CT) imaging to predict recurrence in non-muscle-invasive bladder cancer (NMIBC) patients who underwent transurethral resection of bladder tumor (TURBT).

**Methods:**

A total of 233 patients with NMIBC who underwent TURBT were retrospectively analyzed. Within the intratumoral and peritumoral regions of the venous phase images, 1316 radiomics features were extracted. Feature selection was used to identify a set of top recurrence-associated features within the training cohort. Three models were constructed to predict recurrence for a given patient using Random Forest (RF): Model 1 was based on the radiomics features set from the intratumoral region, Model 2 was based on a combination of intratumoral and peritumoral regions, and Model 3 combined the radiomics features from Model 2 and clinical factors. The three models were then independently tested on internal and external cohorts, and their performance was evaluated. We also employed the bootstrap method on the internal cohort to further validate the performance of the model.

**Results:**

Combining intratumoral and peritumoral regions, Model 2 yielded a higher area under the receiver operator characteristic curves (AUC) than Model 1, with 0.826 AUCs of the training cohort. After adding clinical factors, the predictive performance of Model 3 for postoperative recurrence of NMIBC was further improved, and the AUCs of the training, internal, and external validation cohorts of Model 3 were 0.860 (95% CI: 0.829-0.954), 0.829 (0.812-0.863), and 0.805 (0.652-0.840), respectively (all p>0.05). The bootstrap value of Model 3 on the internal cohort was 0.852. Model 3 stratified patients into high- and low-risk groups with significantly different recurrence-free survival (RFS) (p<0.001).

**Conclusion:**

Radiomic features derived from intratumoral regions can predict the 2-year recurrence risk following TURBT in patients with NMIBC. The predictive performance is further enhanced when combined with radiomic features from peritumoral regions and clinical risk factors.

## 1 INTRODUCTION

Bladder cancer (BCa) is one of the most common malignant tumors of the urinary system [1]. Non-muscle-invasive bladder cancer (NMIBC) confining to mucosal (Ta or Tis) or sub-mucosal connective tissue (T1) accounts for approximately 75% of newly detected cases [2, 3]. Transurethral resection of bladder tumor (TURBT) with postoperative intravesical instillation chemotherapy is the recommended treatment for NMIBC [2, 4, 5]. However, recent studies have found that TURBT is associated with a relatively high risk of postoperative recurrence (70%-80%) and progression to muscle-invasive bladder cancer (MIBC) [2, 4, 5]. Thus, a more aggressive treatment and a closer follow-up for repeat TURBT or salvage cystectomy should be considered for patients with high recurrence and progression risk of BCa. Therefore, preoperative recurrence risk prediction is essential for optimizing management and surveillance.

The 2006 European Organization for Research and Treatment of Cancer (EORTC), the Spanish Urological Club for Oncological Treatment (CUETO), and the European Association of Urology (EAU) scoring model are the guideline-recommended predictive tools to aid decision-making for patients with NMIBC [2, 5]. These models are based on several clinical and pathological factors associated with BCa recurrence, including the number of tumors, tumor size, prior recurrence rate, tumor stage, concurrent carcinoma* in situ* (CIS), pathological grade, and age [2, 4-8]. The main limitation of EORTC is the low number of patients treated with Bacillus Calmette-Guérin (BCG). Thus, the CUETO model has been built to overcome this limitation [6, 7]. On the other hand, EAU NMIBC 2021 scoring model is based mainly on the risk of progression, not recurrence [8].

Radiomics is a promising method for translating computational medical images into mineable data and can combine tumor features and clinical data through feature engineering and machine learning techniques to evaluate tumors non-invasively [9, 10]. Early studies have shown that radiomics is valuable in BCa grading, muscle invasion, differentiation, and recurrence prediction [11-13]. However, these studies extracted and analyzed only the features of the intratumor rather than focusing on the characteristics of the peritumor. In recent years, an increasing number of studies have reported that radiomics can improve the predictive efficiency of tumor prognosis by combining peritumoral and intratumoral regions that contain additional information on the tumor microenvironment (TME), which plays an important role in cancer recurrence and progression [10, 14-17]. The interaction between cancer cells and TME structural components allows the acquisition of an invasive phenotype, allowing cancer cells to spread to distant sites from the primary site via a complex and multistep metastatic cascade.

This study aimed to assess the added value of the peritumoral region in predicting tumor recurrence and to develop a combined model for forecasting the recurrence risk of primary NMIBC within two years after TURBT. A random forest (RF) classifier was applied to train the model and rank the influencing factors. In addition to the clinical factors in the 2006 EORTC and EAU NMIBC 2021 models, we added the neutrophil-to-lymphocyte ratio (NLR) and red blood cell distribution width (RDW) in the combined model according to previous studies.

## 2 MATRIIALS AND METHODS

### 2.1 Study Population

In this study, we retrospectively reviewed data from patients at Medical Center 1 between 2016 and 2020 as the testing and internal validation groups, and patients at Medical Center 2 between 2019 and 2020 as the external validation group. The inclusion criteria for patients were as follows: (1) intraoperative pathology confirmed as NMIBC; (2) patients who underwent initial TURBT; (3) clinical stage Ta or T1, and no clinical metastasis; (4) patients undergoing intravesical instillation chemotherapy and regular cystoscopy; (5) patients who completed follow-up after surgery; and (6) CT performed within 30 days before surgery. Patients with any of the following were excluded: (1) history of cancer; (2) history of radiotherapy and chemotherapy treatment; (3) poor image quality, including but not limited to poor bladder filling and high-density contrast material in the bladder; (4) tumor size: <1 mm; and (5) Incomplete clinical pathology data.

A total of 181 patients from Medical Center 1 represented the primary cohort, which was randomly divided into a training cohort (N =144) and an internal validation cohort (N =37) using a random split-sample (8:2) approach. The external validation cohort comprised 52 patients who fulfilled the selection criteria from the Medical Center 2. A flowchart of the study is shown in Fig. (**1**).

This study was conducted in accordance with the principles of the Declaration of Helsinki. It was approved and monitored by the ethics committees of two medical centers (SWYX: No. 2023-424; No. 2023-05-06). The requirement for informed consent was waived owing to the retrospective nature of the study.

### 2.2 Patient Treatment, Follow-up, and Outcomes

All patients underwent complete transurethral resection of the bladder tumor (TURBT), and complete resection was performed, including the muscle layer of the bladder wall. After TURBT, patients without suspected bladder perforation were administered intravesical instillation of chemotherapy, and additional intravesical chemotherapy with pirarubicin, gemcitabine, or BCG was started 7-15 days after resection.

All patients were followed up once within 3 months after surgery, then every 6 months for the next 2 years, and annually thereafter. Clinical examinations mainly included cystoscopy and imaging examinations [2]. All participants were followed up for at least 2 years. The study endpoint was either cancer recurrence or study termination.

### 2.3 Image Acquisition and Region of Interest (ROI) Delineation

All patients underwent three-phase CT examination. Plain scanning and three-phase dynamic-enhanced scanning were performed. Dynamic enhanced scanning of the arterial, venous, and delayed phases was performed at 30, 60, and 150 s after the bolus-triggering threshold of 120 HU was reached at the thoracoabdominal aorta junction. The CT acquisition settings were further explained in the Supplementary file.

Two radiologists with more than 10 years of clinical experience in abdominal CT interpretation, respectively, manually segmented the CT images in the venous phase using an open-source 3D slicer software (version 5.0.3, www.slicer.org). If multiple lesions were encountered, all lesions were delineated to ensure that complete information about the tumor was obtained. The entire tumor volume (ROI1) and the peripheral ring surrounding the primary tumor region were used to extract features. The peripheral ring automatically dilated the tumor boundaries by 2 mm on the outside and shrunk the tumor boundaries by 1 mm on the inside (a ring with a thickness of 3 mm). Air cavities, adjacent organs, and large vessels were excluded. ROI2 was the tumoral region and peripheral ring Fig. (**2**). In the case of disagreement, data were obtained through discussion. A radiologist randomly selected and re-segmented 30 lesions to calculate the interclass correlation coefficient (ICC).

### 2.4 Image Preprocessing, Feature Extraction and Selection

Image normalization processing is carried out because the research data source has a large time span, and the collection equipment and parameters are different. Pyradiomics 3.0.1 was used to extract the radiomics features based on the original images and images preprocessed by LoG filtering and Wavelet transformation, which could be divided into seven groups, namely first-order statistics, shape features, GLCM features, GLDM features, GLRLM features, GLSZM features and NGTDM features. Image preprocessing and feature extraction were further explained in the Supplementary file. Next, we used variance analysis combined with the least absolute shrinkage and selection operator (LASSO) method with 5-fold cross-validation to reduce the feature dimensions and eliminate redundant and invalid features Fig. (**3**). The signature was obtained by applying linear weighting based on the features selected using the LASSO algorithm.

### 2.5 Model Construction

The selected features were used in the machine learning models. Three models were built by coding in Python language (3.7.11) using RF: Two radiomics-based models were constructed using signatures extracted from ROI 1 (Model 1) and 2 (Model 2). The clinical and pathological parameters, including age, gender, number of tumors, tumor size, tumor stage, pathological grade (WHO 2004/2016 grading classification), NLR, and RDW, were combined with radiomics features to build a clinical-radionics model (Model 3). All models were validated using internal and external test cohorts.

Accuracy, sensitivity, specificity, and AUC were calculated. Important factors in the model were ranked according to the RF classifier. The receiver operating characteristic (ROC) curve of the best-performing model was used to determine the best cutoff value to stratify patients into high- and low-risk recurrence groups. The Youden index was used to select the best cut-off value, where the sum of the sensitivity and specificity was maximized. According to grouping by the cutoff value, recurrence-free survival (RFS) was evaluated using Kaplan-Meier survival plots and verified in internal and external test cohorts.

### 2.6 Statistical Analysis

Statistical analyses were performed using R software version 4.0.4 (R Foundation for Statistical Computing, Vienna, Austria) and IBM SPSS Statistics version 26.0 for Windows (IBM Corporation, Armonk, NY, USA). Continuous data was presented as mean ± standard deviation (SD) or median and interquartile range (IQR) as evaluated with the Student’s t-test or Mann–Whitney U test based on the data distribution. Categorical data were expressed as n (%) and analyzed using the Chi-square test or Fisher’s exact test. Variance analysis combined with LASSO method to select features. RF was used to construct models and rank the important factors. Delong test was used to compare the performance of the models. RFS was evaluated using Kaplan-Meier survival plots, and the log-rank test was used to compare the groups. Bootstrapping (1000 resamples) on the internal cohort (the train and internal validation cohort) was conducted to further evaluate the accuracy of the model. A p-value < 0.05 was considered to be statistically significant.

## 3 RESULTS

### 3.1 Demographic and Clinical Characteristics of Patients

The demographic and clinical characteristics of the patients in the training (N =144), internal validation (N = 37), and external validation (N=52) cohorts are summarized in Table **1**. During follow-up, 44 patients had recurrence (18.9%) and 189 patients had no recurrence within 2 years after TURBT (81.1%). A comparison of the characteristics between the training cohort and validation cohort is shown in Table **2**. The average ICC was 0.897, indicating good consistency among the observers.

### 3.2 Feature Extraction and Selection

A total of 1316 radiomics features were extracted from the tumor volume and peripheral ring, including 252 first-order statistics, 14 shape-based features, 336 GLCM features, 224 GLRLM features, 224 GLSZM features, 196 GLDM features, and 70 NGTDM features.

Using variance analysis combined with LASSO cross-validation, seven features, and five features were filtered out from ROI 1 and ROI 2, respectively Fig. (**4**). The signatures and their coefficients are as follows.

**signature (ROI1)** = wavelet-HHL_glcm_Difference Entropy*(-0.031) + wavelet-LHH_glszm_SizeZoneNonUnifor mity*(0.0329) + log-sigma-1-0-mm-3D_glszm_SizeZoneNon Uniformity*(0.0137) + wavelet-HHL_glcm_DifferenceVarian ce*(-0.025) + wavelet-LHH_glszm_GrayLevelNonUniformity *(0.0318) + wavelet-HHL_glcm_Imc1*(-0.0585) + wavelet-HLH_glszm_SizeZoneNonUniformity*(0.0409) + 0.1475.

**signature (ROI2)** = wavelet-LHL_firstorder_Maxi mum*(0.0173) + wavelet-HLL_glcm_SumEntropy*(-0.0401) + wavelet-HHL_glcm_JointEntropy*(-0.0943) + wavelet-LHL_glszm_SizeZoneNonUniformity*(0.0607) + log-sigma-2 -0-mm-3D_glszm_GrayLevelNonUniformity*(0.0509) + 0.13 32'.

### 3.3 Model Development and Predictive Performance Inspection

**Fig. (5):**
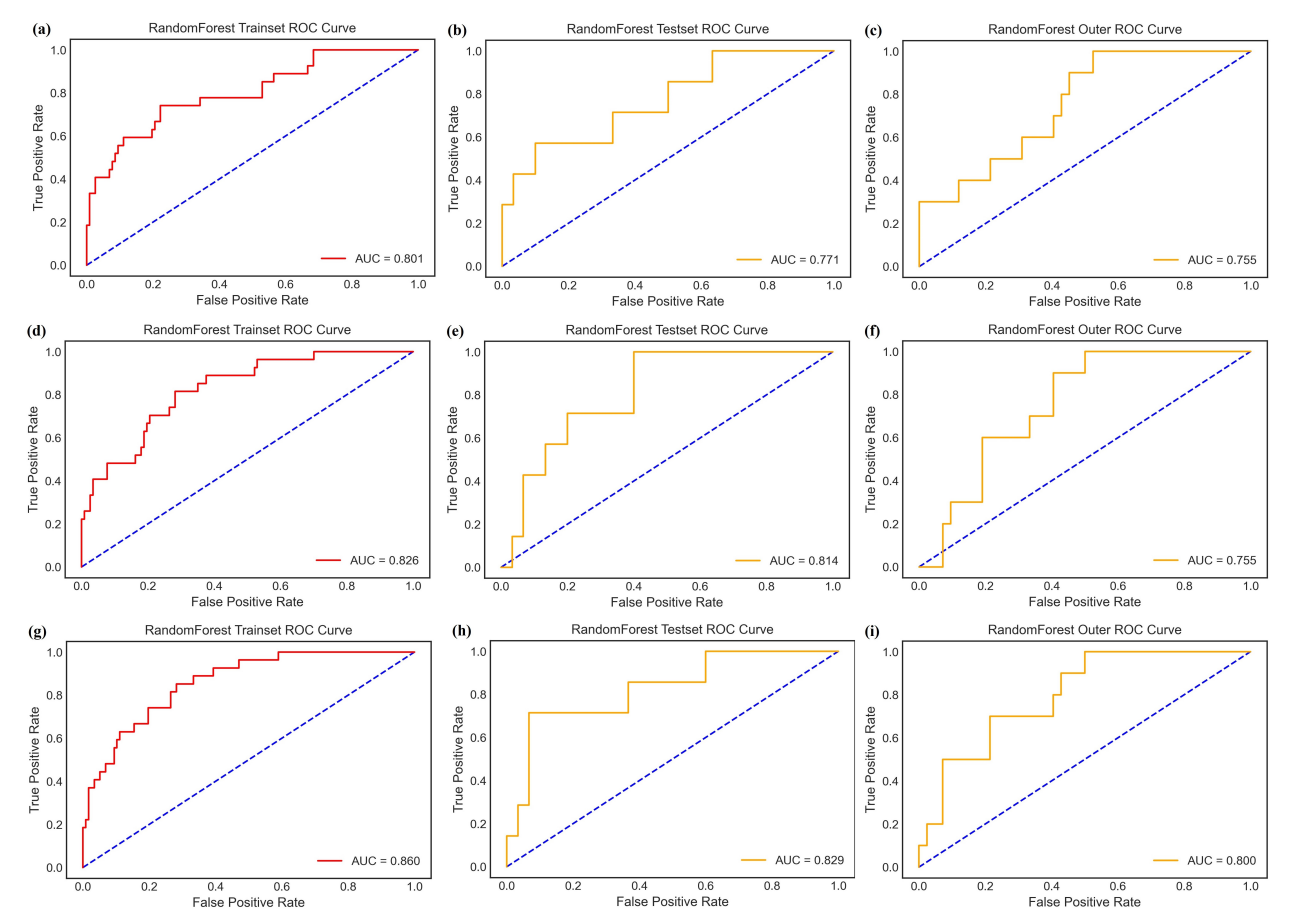
The ROC curve for predicting the recurrence risk of models in each cohort. Model 1: **a-c**; Model 2: **d-f**; Model 3: **g-i**. (a, d, g: training cohort; b, e, h: internal validation cohort; c, f, i: external validation cohort).

The AUC of Models 1 and 2 for predicting the recurrence of NMIBC within 2 years after TURBT were 0.801 (95% CI=0.789-0.916) and 0.826 (95% CI=0.798-0.936) in the training cohort, 0.771 (95% CI=0.751-0.929) and 0.814 (95% CI=0.771-0.858) in the internal validation cohort, and 0.755 (95% CI=0.742-0.814) and 0.755 (95% CI=0.660-0.789) in the external validation cohort, respectively (Table **3** and Fig. **5**). Model 2, which included features from the intratumoral and peritumoral regions, showed better predictive performance (p=0.677 for internal cohort validation and p=0.619 for external cohort validation). The coefficients and correlations of the most important radiomics feature for Model 1 and Model 2 are shown in Fig. (**4**). These findings indicated that the signatures from the combined region were more useful in predicting recurrence and were used for the subsequent construction of the radiomics-clinical model (Model 3).

The AUC of Model 3 for predicting NMIBC recurrence within 2 years after TURBT was 0.860 (95% CI=0.829-0.954) in the training cohort, 0.829 (95% CI=0.812-0.863) in the internal validation cohort, and 0.805 (95% CI=0.652-0.840) in the external validation cohort (Table **3** and Fig. **5**). The bootstrap value of Model 3 on the internal cohort was 0.852. After adding the clinical factors, the model prediction performance was further improved. The order of importance of the most important radiomics features for Model 3 is shown in Fig. (**6**).

Delong’s test detected no statistical differences among the three models (all p>0.05, Table **4**). However, the probability of Model 3 obtained using RF revealed a significant difference between the recurrence and non-recurrence groups (p<0.05) Fig. (**7**). The patients were divided into two subgroups according to the cut-off value of 0.454. The training cohort Kaplan-Meier survival plot factored by cut-off value was performed, revealing a significant difference (p<0.001) in mean RFS, as was the internal and external test cohorts Fig. (**8**).

## 4 DISCUSSION

Bladder cancer has the highest lifetime treatment costs of all cancers [18, 19]. This is due to high recurrence rates in the large group of NMIBC patients, and associated intensive surveillance strategies. Repeated TURBT or salvage RC for recurrent patients will cause poor prognosis and incur greater treatment costs [20]. Therefore, a highly effective preoperative recurrence risk prediction model that will benefit from less intensive surveillance schedules will both mitigate the healthcare costs of surveillance and the burden on the individual patient and would be essential for optimizing management. At present, the EORTC risk tables, the CUETO, and the EAU scoring model are widely used in clinical practice, but their prediction performance is poor [21, 22]. In this study, a preoperative and low-cost radiomics analysis was used to integrate imaging features from intratumoural and peritumoral regions on CT images to predict NMIBC recurrence risk after TURBT.

We developed three models, all of which achieved good prediction performance. Model 2, in which both intratumoral and peritumoral regions were combined, performed better than Model 1, which was constructed using the intratumoral region only, thus confirming that the inclusion of peritumoral segmentations improved the efficacy of the models’ performance in predicting recurrence in patients with NMICB post-TURBT. On this basis, we established a radiomics-clinical combined model to further improve predictive efficiency. The combined model showed optimal performance in predicting NMIBC recurrence within 2 years after TURBT. When the model suggests a high risk of recurrence, more aggressive treatment and postoperative surveillance should be taken.

Recently, studies on radiomics have enhanced our understanding of the role of radiomics in distinguishing tumor homogeneity and heterogeneity [23]. On the other hand, an increasing number of studies have highlighted the vital role of TME in cancer prognostic and clinical outcomes [17, 24]. The TME, which refers to the cellular environment wherein tumors or cancer stem cells exist, includes various immune and stromal cells. Previous studies have confirmed the role of radiomics in evaluating TME, such as NLR in TME and tumor stroma status [10, 25]. In this study, model 1, which was based on intratumor features only, also showed a relatively good performance in predicting NMIBC recurrence (AUC=0.801 in the training cohort).

Model 2 was established by combining intratumoral and peritumoral regions, and the prediction effect was improved in both the training and verification cohorts. Many studies in other tumor entities have shown that the incorporation of intratumoral and peritumoral regions can improve the predictive effectiveness of the model, such as glioblastoma [26], hepatocellular carcinoma [15], breast cancer [14], BCa [16], and lung cancer [27]. The peritumoral regions in previous studies mostly included an area around the tumor of 5mm or more, which included a number of macroscopic signs around the tumor, including neovascularization, edema, and spiculation around the tumor. In fact, the peritumoral area also comprises the tumor-stroma interface, and the peritumoral segmentations might therefore, capture important additional information from the surrounding stromal inflammation and immune infiltration. Xue *et al.* [28] analyzed TME subtypes of 189 samples collected from patients and mouse models and observed that GZMK+ CD8+ T cells were mainly localized in the stroma. Huang *et al.* [10] reported an association between NLR of the peritumoral segmentations with the prognosis and the response to gastric cancer immunotherapy. Incorporating intratumoral and peritumoral regions can enhance the understanding of cancer biology and characterization of spatial heterogeneity.

The eight selected clinical and pathological variables for the developed combined model were age, gender, number of tumors, tumor size, tumor stage, pathological grade, NLR, and RDW. Except for age, RDW and NLR were the two important clinical factors in ranking variables obtained using the random forest algorithm. RDW, which reflects the size variability of red blood cells and is a routinely available marker of the systemic inflammatory response, was recently linked to the poor prognosis of various tumors, including NMIBC [29]. Fukuokaya *et al.* [29] reported a significant association between high RDW and shorter time to recurrence (TTR) in patients with NMIBC after TURBT. NLR is another standard hematologic marker reflecting inflammation that has also been reported as a tumor immune status indicator [30]. Several studies have also suggested peripheral blood NLR as an important predictor of clinical prognosis and response to treatment in various malignancies [30-32]. Getzler *et al.* [33] found an increased performance by adding the EORTC score to NLR. Our results also suggested that RDW and NLR were important predictors, which is consistent with the above studies. According to EORTC and CUETO [5, 21], the most important prognostic factors for recurrence are prior recurrence rate, number of tumors, age, gender and grade. However, the contribution of tumor stage and pathological grade in Model 3 changed owing to the addition of radiomics features in this study. All three models were validated on the external validation set, with AUC of 0.755, 0.755, and 0.805, respectively, indicating that the models exhibited predictive power. Model 3 had the best performance in prediction efficiency. It is also worth noting that Delong’s test detected no statistical differences among the three models. Whether the advantage trends of the predictive performance of Model 3 will be significant in a larger sample needs further verification.

CT urography is crucial for the initial investigation of hematuria. Imaging of the upper tract is essential for 2% of cases diagnosed with BCa with an upper tract tumor [34], and the 2022 NCCN guidelines recommend CT urography as the preferred approach to upper tract imaging [4]. Unfortunately, conventional CT images cannot be used to evaluate muscle invasion in BCa because of their unsatisfactory soft tissue resolution. However, CT-based radiomics has enabled deep mining of the biological nature of CT images and comprehensive, noninvasive, and quantitative observation, and it has been reported to be applied in BCa staging, recurrence prediction, and treatment response [11, 12]. Moreover, with the development of AI and computer-aided detection (CAD), significant efforts have been made to develop automated segmentation methods to eliminate inter-segmenter and intra-segmenter (re-test) variability to a certain extent [35].

The present study had some limitations. First, the sample size was relatively small, and data from multiple centers were required to validate the overall performance of the model. In the future, we will continue to collect multi-center and amounts of clinical data to verify the model. Second, other potentially useful predictive factors, such as C-reactive protein (CRP), Ki67, and other clinical and histopathological markers, are also prognostic factors of NMIBC [13], but we did not add this indicator to the study for incomplete data collection due to incomplete data. Third, some practical problems, such as cost-effectiveness and integration with current clinical systems, should be considered. Finally, an increasing number of studies have highlighted that MRI has an essential role in the staging of the bladder due to its increased soft tissue contrast revolution [30]. Nonetheless, further studies are required to establish how well the peritumoral region in MRI imaging predicts recurrence needs. Therefore, the present study requires further improvement and optimization.

## CONCLUSION

Our findings preliminarily demonstrate that radiomic features derived from intratumoral regions can predict the 2-year recurrence risk following TURBT in patients with NMIBC. The predictive performance is further enhanced when combined with radiomic features from peritumoral regions and clinical risk factors. The extent of this improvement in predictive efficacy still needs to be validated in larger samples to aid in the early clinical identification of patients at high risk of recurrence, thereby guiding the development of personalized treatment plans in the future.

## AUTHORS’ CONTRIBUTIONS

X.W.: Study conception and design; L.L., N.L., C.G.: Data collection; L.C.: Analysis and interpretation of results; T.C.:Draft manuscript; H.Z., K.W.: Methodology; W.S.: Visualization.

## Figures and Tables

**Fig. (1):**
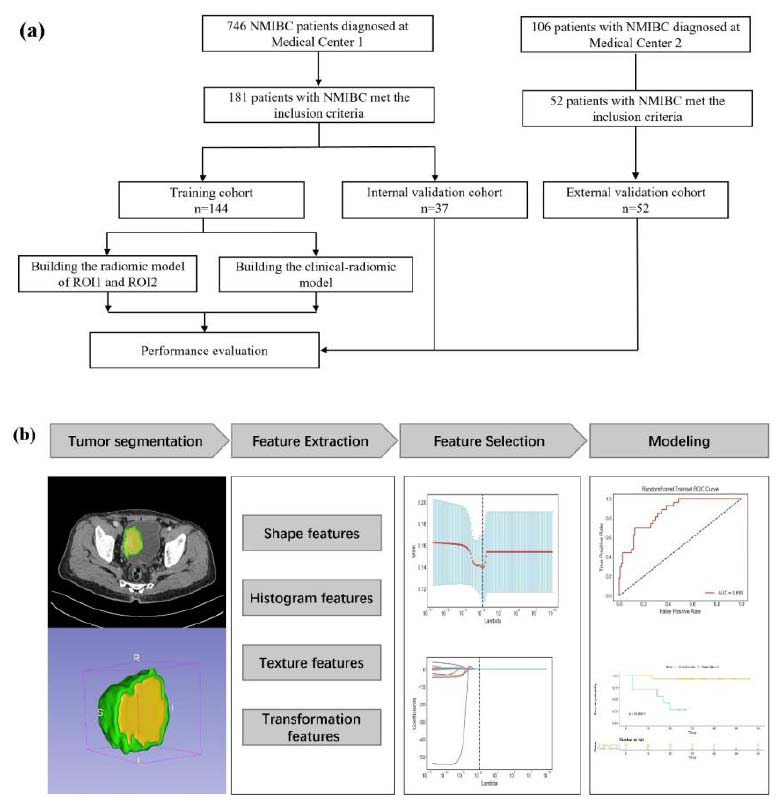
Flowchart of patients (**a**) and study design (**b**). NMIBC: non-muscle-invasive bladder cancer NMIBC: non-muscle-invasive bladder cancer.

**Fig. (2):**
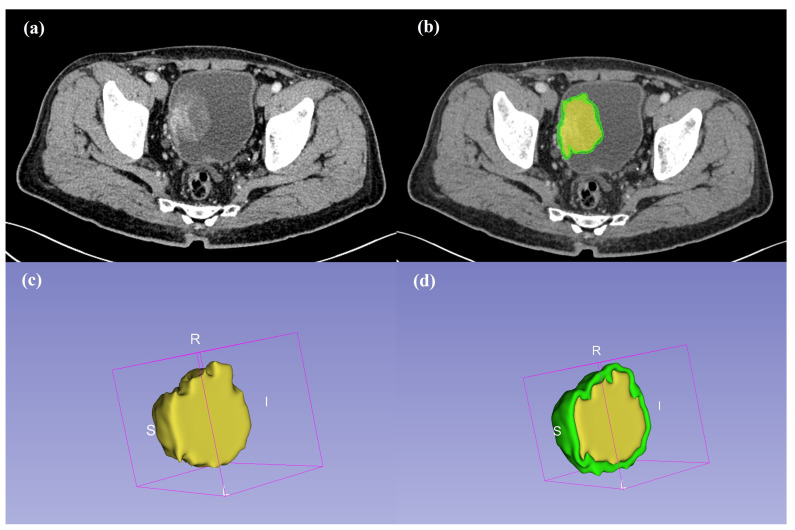
**(a)** Venous phase images with tumor. **(b)** Two regions enclosing the intratumoral (yellow) and peritumoral ring (green). **(c,d)** 3D-VOI of the intratumor region (ROI 1, c) and the intratumoral region, and the peripheral ring (ROI 2, d).

**Fig. (3):**
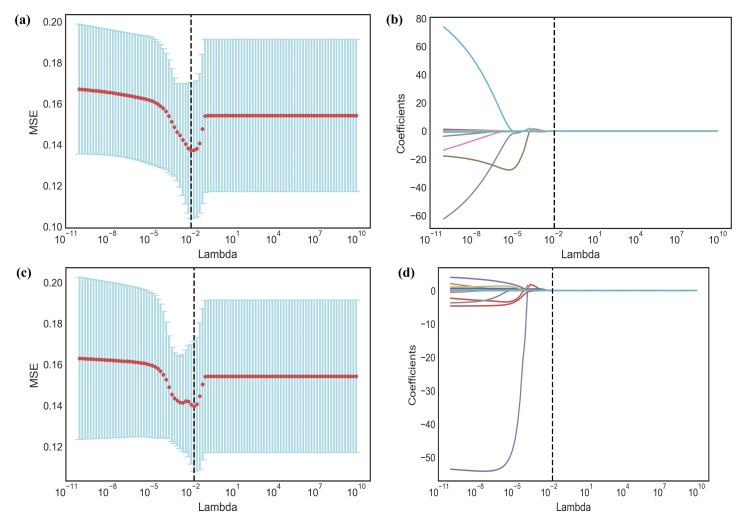
Identification of the significant radiomic features associated with recurrence using the least absolute shrinkage and selection operator (LASSO) method. ROI 1 (**a, b**); ROI2 (**c, d**).

**Fig. (4):**
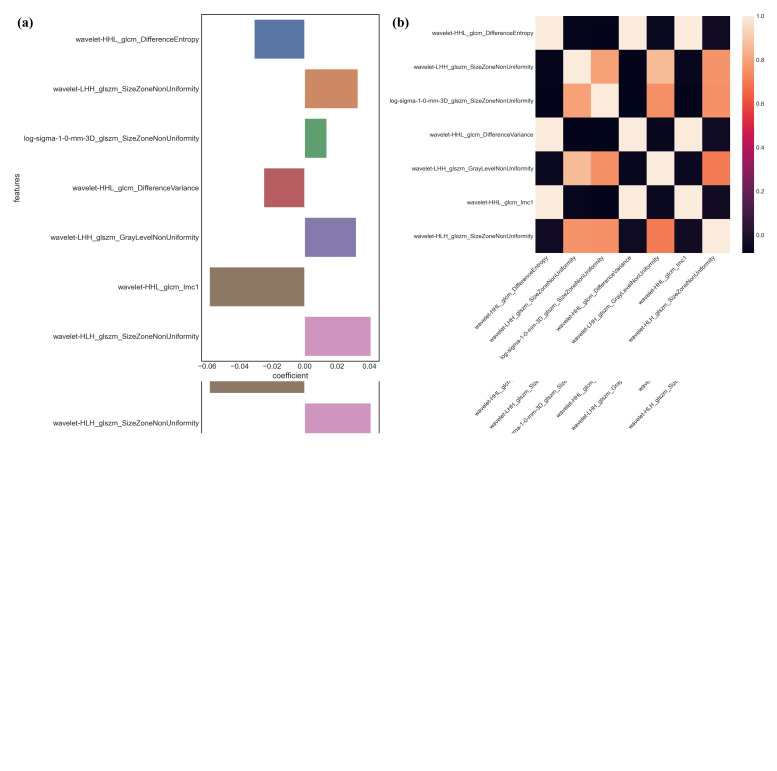
The coefficient and correlation of the most critical radiomics features for Model 1 **(a, b)** and Model 2 **(c, d)**.

**Fig. (6):**
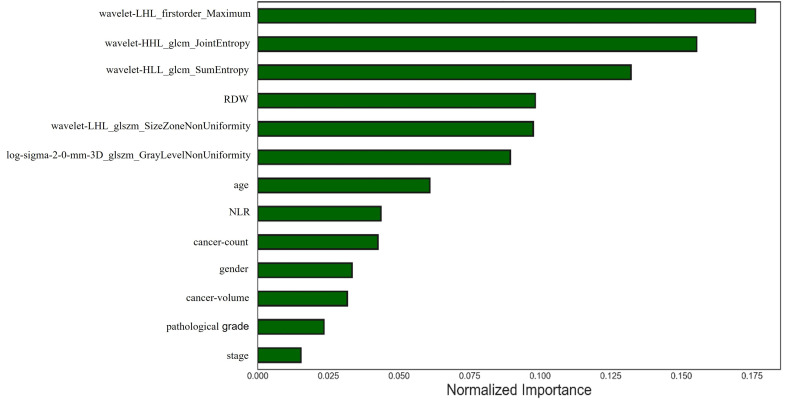
The order of the most important radiomics features for Model 3.

**Fig. (7):**
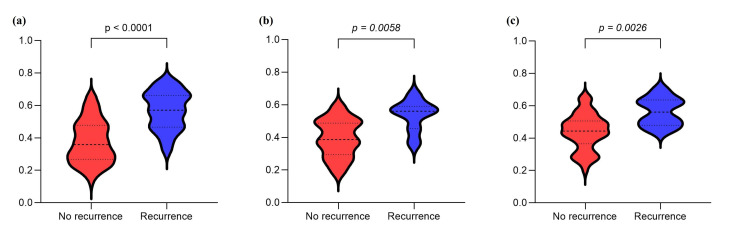
The distribution of the probability between the recurrence and nonrecurrence groups. **(a)** Training cohort; **(b)** Internal validation cohort. **(c)** External validation cohort.

**Fig. (8):**
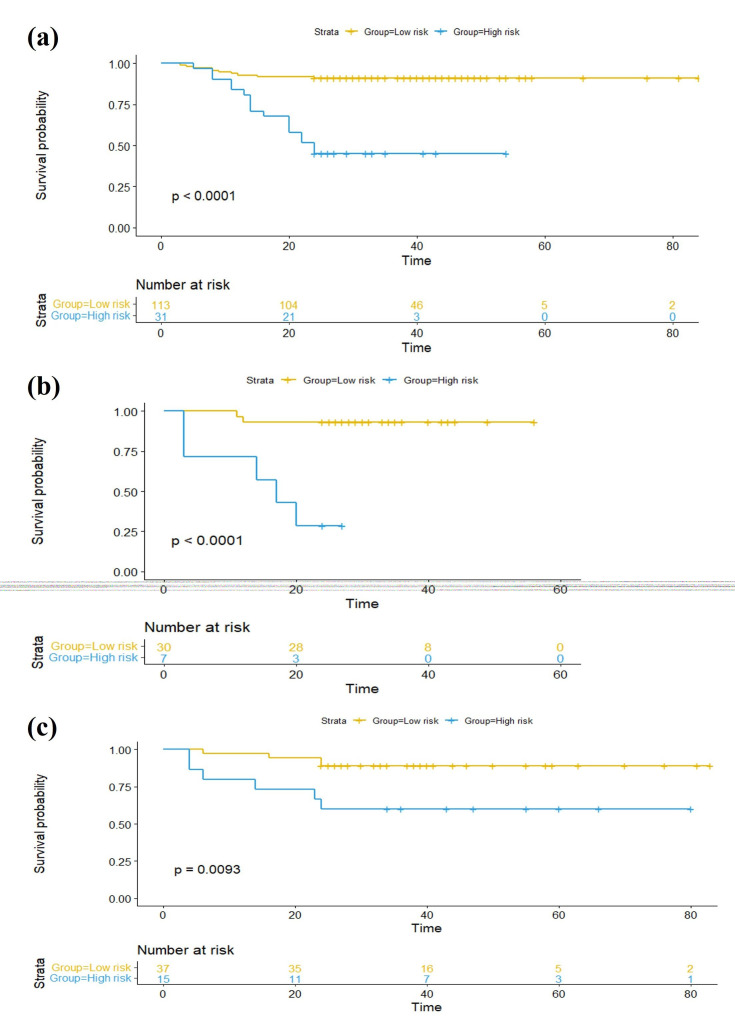
Kaplan–Meier plots of NMIBC within 2 years after surgery in each cohort. **(a)** training cohort; **(b)** internal validation cohort; **(c)** external validation cohort.

**Table 1:** Characteristics of patients with NMIBC in each cohort.

	Training Cohort (n=144)		Internal Validation Cohort (n=37)			External Validation Cohort (n=52)	
n	%	n		%	n	%
**Gender**							
Male	120	83.3	28		75.7	43	82.7
Female	24	16.7	9		24.3	9	17.3
Age (years)	65.5 [57.8; 72.0]		66.0 [60.0; 74.0]			66 [58.0; 78.0]	
**Grade**							
Low	81	56.3	21		56.8	18	34.6
High	63	43.8	16		43.2	34	65.4
**Staging**							
Ta	65	45.1	18		48.6	24	46.2
T1	79	54.9	19		51.4	28	53.8
**Number of tumors**							
Single	109	75.7	27		73.0	35	67.3
Multiple	35	24.3	10		27.0	17	32.7
**Tumor size**							
<3cm	96	66.7	28		75.7	26	50.0
≥3cm	48	33.3	9		24.3	26	50.0
**NLR**	1.93[1.49; 2.75]		1.94[1.58; 2.62]			2.08[1.56; 2.85]	
**RDW(fl)**	42.2[40.1; 44.7]		43.4[40.4; 45.2]			42.9[40.5;44.6]	
**Label**							
Recurrence	27	18.8	7	18.9		10	19.2
No recurrence	117	81.2	30	81.1		42	80.8

**Abbreviations:** *NLR:Neutrophil-to-Lymphocyte ratio;RDW:Red cell distribution width.

**Table 2:** The comparison of characteristics between the training cohort and validation cohort.

**Characteristics**	**Training *vs.* Internal validation** **p-value**	**Training *vs.* External validation** **p-value**
Gender (Male, Female)	0.482	0.916
Age (years)	0.817	0.120
Pathological Grade (Low, High)	0.956	0.080
Stage (Ta, T1)	0.703	0.900
Number of tumors (Single, Multiple)	0.733	0.242
Tumor size (<3cm,≥3cm)	0.294	0.034
NLR	0.997	0.051
RDW (fl)	0.742	0.864
Recurrence (yes, no)	0.981	0.940

*NLR:Neutrophil-to-Lymphocyte ratio;RDW:Red cell distribution width.

**Table 3:** The diagnostic performance of models in each cohort.

	Model 1			Model 2			Model 3		
Training cohort	Internal validation cohort	External validation cohort	Training cohort	Internal validation cohort	External validation cohort	Training cohort	Internal validation cohort	External validation cohort
Specificity	0.778	0.767	0.690	0.795	0.733	0.690	0.812	0.800	0.738
Sensitivity	0.704	0.571	0.500	0.704	0.714	0.600	0.667	0.714	0.700
Accuracy	0.764	0.730	0.654	0.778	0.730	0.673	0.785	0.784	0.731
AUC (95% CI)	0.801 (0.789-0.916)	0.771 (0.751-0.929)	0.755 (0.742-0.814)	0.826 (0.798-0.936)	0.814 (0.771-0.858)	0.755 (0.660-0.789)	0.860 (0.829-0.954)	0.829 (0.812-0.863)	0.805 (0.652-0.840)

**Table 4:** P-values of the three models obtained by Delong’s test.

	Variable 1	Variable 2	Z	P
Internal validation cohort	Model 1	Model 2	-0.416	0.677
	Model 1	Model 3	-0.539	0.590
	Model 2	Model 3	-0.251	0.802
External validation cohort	Model 1	Model 2	-0.497	0.619
	Model 1	Model 3	-0.497	0.619
	Model 2	Model 3	0.000	1.000

## Data Availability

The data and supportive information are available within the article.

## LIST OF ABBREVIATIONS

EORTC: European Organization for Research and Treatment of Cancer
EAU: European Association of Urology
BCa: Bladder cancer
TURBT: Transurethral resection of bladder tumor
NMIBC: Non-muscle-invasive bladder cancer
CT: Computed tomography
RF: Random Forest
RFS: Recurrence-free survival
TME: Tumor microenvironment
TTR: Time to recurrence
CAD: Computer-aided detection
CRP: C-reactive protein

## STANDARDS OF REPORTING

STROBE guidelines were followed.
