# Supplementary material for: CT-based Radiomics of Intratumoral and Peritumoral Regions to Predict the Recurrence Risk in Patients with Non-muscle-invasive Bladder Cancer within Two Years after TURBT
Source: Curr Med Imaging. 2025 May 26;21:e15734056350444. doi: 10.2174/0115734056350444250418075406 (PMC13176758; doi:10.2174/0115734056350444250418075406)
Supplement: Supplementary file 1 — Supplementary material is available on the publisher’s website along with the published article. [file CMIM-21-E15734056350444_SD1.pdf]

1573-4056/25

Send Orders for Reprints to reprints@benthamscience.net

BENTHAM SCIENCE

CrossMark

Current Medical Imaging

Content list available at: https://benthamscience.com/journals/cmir

Supplementary Material

CT-based Radiomics of Intratumoral and Peritumoral Regions to Predict the Recurrence Risk in Patients with Non-muscle-invasive Bladder Cancer within Two Years after TURBT

Ting Cao[1,3], Na Li[2], Chuanchao Guo[3], Hepeng Zhang[4], Lihua Chen[5], Ke Wu[3], Lisha Liang[3], Ximing Wang[6] and Wen Shen[5,*]

[1]*Department of Radiology, First Central Clinical College, Tianjin Medical University, Tianjin, China*
[2]*Department of Radiology, The People's Hospital of Zhangqiu Area, Jinan, China*
[3]*Department of Radiology, The Affiliated Taian City Central Hospital of Qingdao University, Taian, China*
[4]*Department of Urology Surgery, The Affiliated Taian City Central Hospital of Qingdao University, Taian, China*
[5]*Department of Radiology, Tianjin First Central Hospital, School of Medicine, Nankai University, Jinan, China*
[6]*Department of Radiology, Shandong Provincial Hospital Affiliated to Shandong First Medical University, Jinan, China*

1.1. CT Image Acquisition

Images were collected from six CT machines at two medical centers: Siemens CT scanner (SOMATOM Definition Flash and SOMATOM Force), TOSHIBA CT scanner (Aquilion One 640), and Philips CT scanner (Ingenuity CT, Royal Philips) at Medical Center 1; Siemens CT scanner (SOMATOM Definition AS) and Philips CT scanner (Ingenuity CT, Royal Philips) at Medical Center 2. The following scanning parameters were applied: tube voltage of 120 kVp, automatic tube current, FOV 500 mm, a scanning layer thickness of 1-1.5mm, and the interlamellar spacing of 1 mm. For enhanced scanning, iodine contrast medium (350 mg I/ml) was injected via the elbow vein at an injection rate of 2.5-3 ml/s, and plain scanning and three-phase dynamic enhanced scanning were performed. The dynamic enhanced scanning of the arterial, venous, and delay phases was performed after 30, 60, and 150 s, respectively, after the bolus-triggering threshold of 120 HU had been reached at the thoracoabdominal aorta junction, respectively.

1.2. Image Preprocessing and Feature Extraction

After optimization and debugging, the bin width of the histogram is set to 25. This parameter affects the gray level discretization process and is used to compute texture features. A larger bin width can reduce the amount of computation, but some texture information may be lost. We tested different values and got the best one.

The pixel spacing after resampling was set to 3*3*3 mm. This parameter is used to adjust the resolution of the original image to a uniform resolution to eliminate the effect of different image resolutions on feature extraction. Because there is no relevant research to prove the value method of this parameter, the optimal value is obtained through the same test.

Since the research data sources had a large time span, and the acquisition equipment and parameters were different, image normalization was performed.

Pyradiomics 3.0.1 was used to analyze the original images, five log-filtered images (sigma = 1.0, 2.0, 3.0, 4.0, 5.0) and Wavelet transform images. The Wavelet transform produced eight subbands (LLL, LLH, LHL, LHH, HLL, HLH, HHL, HHH). For each image type (raw, LoG, Wavelet), we extracted the above seven categories of features. By default, the number of features was as follows:

First Order Statistics: 18; Shape-based features: 14; GLCM features: 24; GLRLM features: 16

GLSZM features: 16; GLDM features: 14; NGTDM features: 5

The number of features was as follows:

Original image: 18 (First Order) + 14 (Shape) + 24 (GLCM) + 16 (GLRLM) + 16 (GLSZM) + 14 (GLDM) + 5 (NGTDM) = 107

LoG images (per sigma): 18 (First Order) + 24 (GLCM) + 16 (GLRLM) + 16 (GLSZM) + 14 (GLDM) + 5 (NGTDM) = 93

DOI: 10.2174/0115734056350444250418075406, 2025, *21*, e15734056350444

Wavelet-transformed image (each subband): 93 features

The total number of features:

Original image: 107

LoG image: 5 * 93 = 465

Image after Wavelet transform: 8 * 93 = 744

The total number of features was 107 + 465 + 744 = 1316

So, based on the given Settings, 1316 features were extracted.

© 2025 The Author(s). Published by Bentham Science Publisher.

This is an open access article distributed under the terms of the Creative Commons Attribution 4.0 International Public License (CC-BY 4.0), a copy of which is available at: https://creativecommons.org/licenses/by/4.0/legalcode. This license permits unrestricted use, distribution, and reproduction in any medium, provided the original author and source are credited.
